# Bipolaron Dynamics in Graphene Nanoribbons

**DOI:** 10.1038/s41598-019-39774-2

**Published:** 2019-02-27

**Authors:** Gesiel Gomes Silva, Luiz Antonio Ribeiro Junior, Marcelo Lopes Pereira Junior, Antonio Luciano de Almeida Fonseca, Rafael Timóteo de Sousa Júnior, Geraldo Magela e Silva

**Affiliations:** 1 Goiás Federal Institute of Education, Science and Technology, Luziania, Goias 72.811-580 Brazil; 2 Department of Physics, Chemistry and Biology (IFM), Linköping University, SE-581 83 Linkoping, Sweden; 3 International Center for Condensed Matter Physics, University of Brasília, P.O. Box 04513, 70910-900 Brasília, Brazil; 4 University of Brasília, PPG-CIMA, Campus Planaltina, 73345-010 Brasília, DF Brazil; 5 Institute of Physics, University of Brasília, Brasília, 70910-900 Brasília, Brazil; 6 Department of Electrical Engineering, University of Brasília, CP04455, Brasília, 70919-970 Brazil

## Abstract

Graphene nanoribbons (GNRs) are two-dimensional structures with a rich variety of electronic properties that derive from their semiconducting band gaps. In these materials, charge transport can occur via a hopping process mediated by carriers formed by self-interacting states between the excess charge and local lattice deformations. Here, we use a two-dimensional tight-binding approach to reveal the formation of bipolarons in GNRs. Our results show that the formed bipolarons are dynamically stable even for high electric field strengths when it comes to GNRs. Remarkably, the bipolaron dynamics can occur in acoustic and optical regimes concerning its saturation velocity. The phase transition between these two regimes takes place for a critical field strength in which the bipolaron moves roughly with the speed of sound in the material.

## Introduction

Graphene nanoribbons are two-dimensional honeycomb-like lattices formed by laterally confined semiconducting strips of graphene sheets^1^. Since GNRs can present semiconducting energy gaps of atomically precise control^2^, they represent a promising scaffold in exploring the charge transport properties in the next-generation organic optoelectronic materials. One of the critical aspects that can impact the performance of GNR-based devices is the formation and subsequent transport of charge carriers. Nevertheles, such mechanisms concerning GNRs remain not entirely understood. Also, the nature of the quasiparticles that are possible to rise in these materials was not wholly revealed.

Recently, some experimental^3–7^ and theoretical^8–13^ studies — including our previous researches^14–18^ — have addressed the charge transport mechanism, along with its underlying properties, in GNRs. Bischoff and coworkers have pointed out that, for the electrical transport experiments in GNRs, there are in the literature different interpretations of similar findings^3^. However, there is a consensus about the strong dependence of the coupling strength to neighboring states in forming localized charge carriers. Theoretical investigations, based on tight-binding models, show that zig-zag GNRs are always metallic with the presence of sharply localized edge states for charge carriers^19^. Contrarily, Modarresi *et al*. have proposed a semiconducting-like behavior for the charge transport mechanism in zig-zag GNRs through the possible polaron formation in the presence of Rashba spin-orbit coupling^8^. It is worthwhile to mention here that, in organic low-dimensional systems, polarons are self-localized electronic states yielded due to their strong electron-lattice interactions. Moreover, in some previous works of our research group, it was demonstrated that polarons are the immediate quasiparticle solutions in the armchair GNRs when electrons are removed from or added to the lattice^14,20^. The works as mentioned earlier point to the importance of charge localization for the transport phenomena in GNRs. The interpretation of the polaron concept is strictly dependent on the system in which this kind of quasiparticle arises. Very recently, experimental results have revealed the generation of polarons in the interface of graphene/hexagonal boron nitride (h-BN) van der Waals heterostructures (vertical stacks composed of these layered materials)^21^. In that study the authors have observed zone-corner replicas of h-BN valence band maxima, with energy spacing coincident with the highest phonon energy of the heterostructure, an indication of Fröhlich polaron formation due to forward-scattering electron-phonon coupling^21^. In other words, in graphene/hexagonal heterostructures the charge of the polaron and related deformations are vertically distributed between the lattice structures that form the interface in such a way that its lattice deformations that are associated with the presence of charge consider both intra and interchain distortions. Importantly, different structural arrangements for this kind of quasiparticle may lead to distinct charge transport mechanisms^22,23^.

In conducting polymers, for instance, the conventional self-localized charge carriers are polarons that have spin 1/2 and charge ±*e*^24^. However, it is well accepted that that bipolarons can be formed in in these materials from a large concentration of polarons^24^. In this picture, two acoustic polarons with the same charge and antiparallel spins can recombine to form an acoustic bipolaron. Consequently, bipolarons are spinless structures with charge ±2*e*. Since some optoelectronic processes can conduct to the formation of bipolarons in other classes of organic materials, it is plausible to expect their creation in GNRs and this work is aimed to investigate such a process.

Herein, the possible bipolaron formation in armchair GNRs is theoretically investigated using a two-dimensional tight-binding approach that includes lattice relaxation effects and Hubbard electron-electron interaction terms. In the present work we numerically study the ground state and dynamical properties for this species of charge carrier concerning different GNR families and electric field strengths. The yielded results for the bipolaron properties in GNRs are qualitatively similar to those obtained in the cases for conducting polymers. This fact suggests that bipolarons may assist the charge transport mechanism in GNRs.

## Results

**Figure 1:**
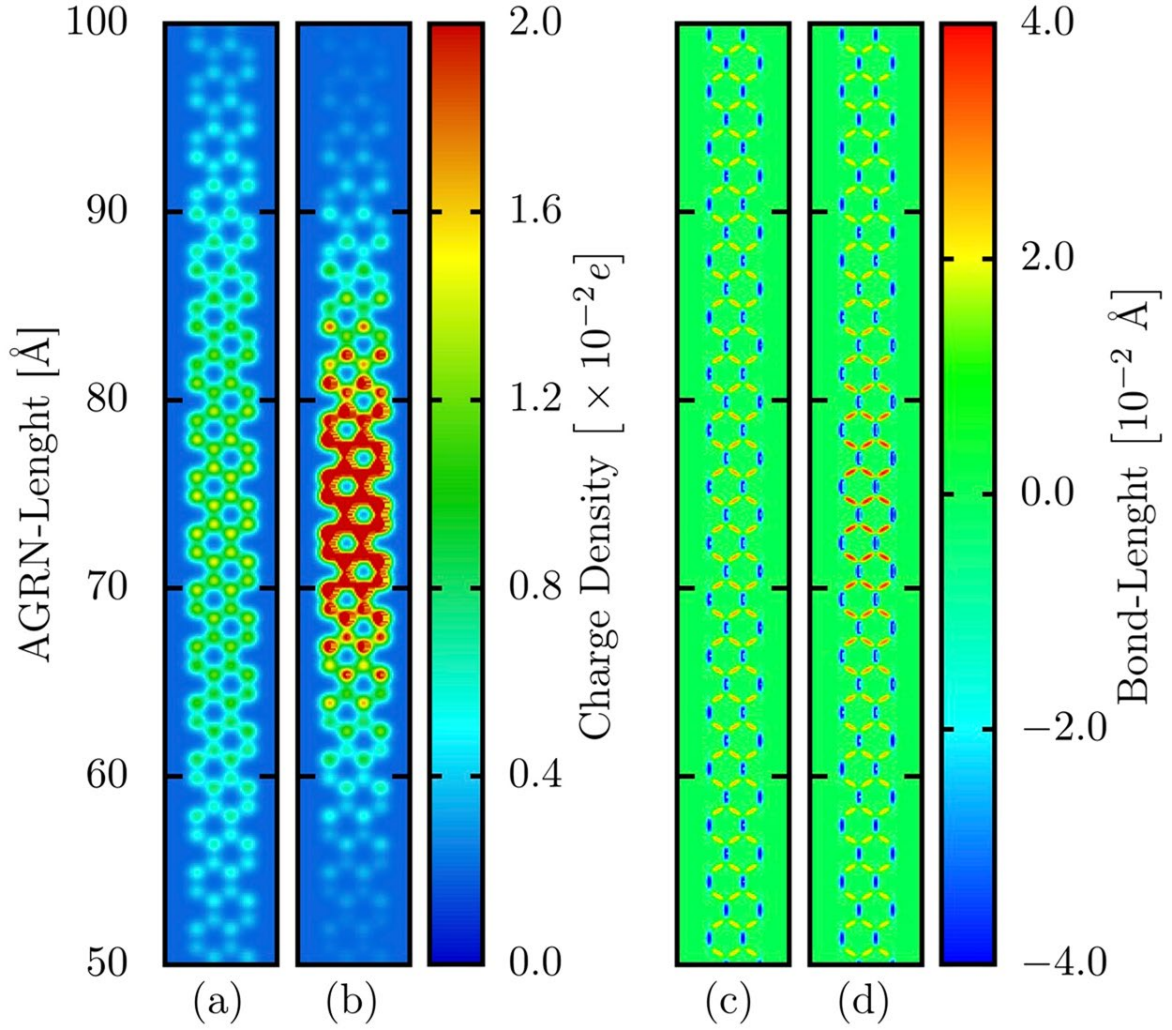
(**a**,**b**) Charge density and (**c**,**d**) bond-length profiles for a lattice containing (**a**,**c**) a polaron and (**b**,**d**) a bipolaron. These panels zoom-in the region where the charge is localized for an ANGR–4 with 300 Å of length.

We begin the discussions by presenting the initial state properties of a bipolaron in an armchair GNR with four atoms width (AGNR-4). For the sake of comparison, Fig. 1(a,c) depict the charge localization and the bond-length patterns for a system including a polaron whereas Fig. 1(b,d) refer to these patterns for a bipolaron endowed lattice. These panels zoom-in the region where the charge is localized for an AGNR–4 with 300 Å of length. In Fig. 1(a,b), one can note that the AGNR containing a bipolaron presents a higher degree of charge localization (signatures in red). This quasiparticle have a similar extension to the polaron, approximately 30 Å. Remarkably, the bipolaron is spontaneously generated once the extra charges have a suitable spin configuration. It suggests that bipolarons are natural quasiparticle solutions in GNRs. To obtain the charge density profile as shown in Fig. 1(b), one electron with spin up and one with spin down were removed. This behavior for the charge localization directly impacts the lattice bond-length pattern. Figure 1(d) shows that the deformations for the bond elongations and compressions, in the region where the excess of charge is localized, are more pronounced in the bipolaron case (red and dark blue regions). These lattice deformations also appear in the polaron case, but in a weaker fashion, as illustrated in Fig. 1(c). Excepting the region containing the charge, the lattice deformations for both cases are similar. Such an interdependent behavior between excess of charge and lattice deformations is clear evidence of forming an ordinary quasiparticle in organic materials^24^. It is worthwhile to mention that the refs^14,20^ discuss in detail the polaron properties in AGNRs.

**Figure 2:**
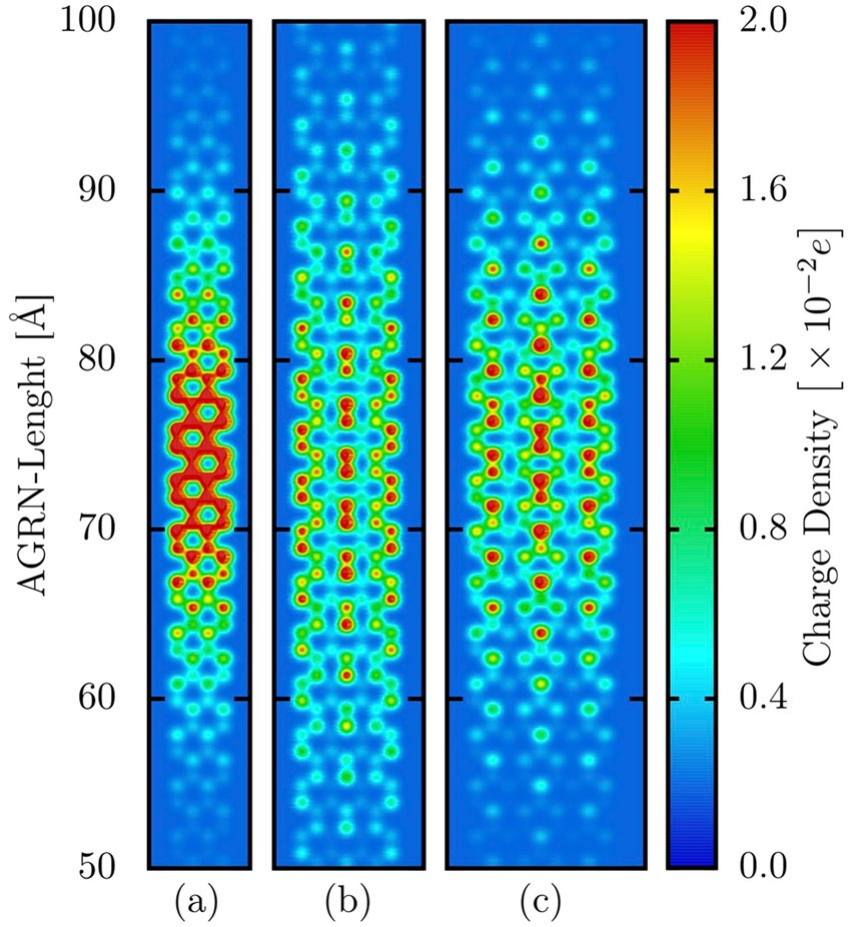
Bipolaron localization in the (**a**) AGNR–4, (**b**) AGNR–7, and (**c**) AGNR–9 lattices.

It was already established that different GNR families present distinct optoelectronic properties^19,25^. An AGNR–*N* is metallic if *N* = 3*p* + 2 (where *p* is a positive integer) otherwise it is a semiconducting material^19^. Since the AGNR width can play the role of altering the quasiparticle properties, it is important to highlight its effect on the charge localization. Figure 2(a–c) illustrates the bipolaron localization in the AGNR–4, AGNR–7, and AGNR–9 lattices, respectively. These lattices belong to the same family, namely, 3*p* + 1 and 3*p*. For the sake of clarity, we show one more time the charge localization for the AGNR–4 lattice. In Fig. 2 one can realize, by observing the color scheme, that the narrower the AGNR width the grater is the charge localization. Moreover, the charge tends to concentrate in the middle of the nanoribbon for narrower AGNRs. For the wider AGNRs, the charge lies, mostly, in three separated regions: a central vertical line and in two vertical armchair lines in the edges. This specific pattern for the quasiparticle localization states that the charge concentrates, alternately, over the carbon-carbon bonds with low and high charge densities. In this way, even in wider AGNRs, the bipolaron formation takes place. Importantly, wider AGNRs tends to lower the impact of the lattice relaxation effects in forming the charge carriers. Therefore, this kind of nanoribbon tends to present small local concentrations and more uniform charge distribution for the excess charge. In an upper critical limit for the AGNR width, the electron-lattice interactions do not lead to charge localization, i.e., no quasiparticle is formed. In this physical picture, the system presents a metallic behavior concerning the charge transport mechanism. Conversely, in the lower critical regime, the lattice relaxation effects become dominant in a such a way that the charge transport mechanism approaches to the one for the conducting polymers, such as polyparaphenylene^26^. It worth to mention here that the bipolaron localization in AGNRs belonging to the family 3*p* are similar to the polaron localization in these lattices, as described in ref.^20^.

**Figure 3:**
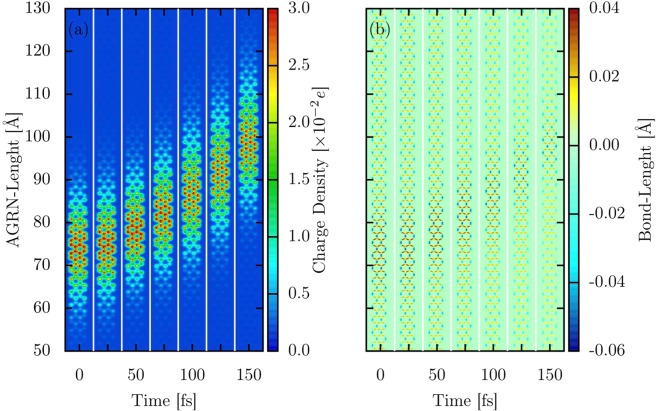
Time evolution for the bipolaron’s mean charge density and bond-length in an AGNR–4 for an electric field strength of 1.0 mV/Å.

We now discuss the bipolaron dynamics in the presence of an external electric field. To do so, Fig. 3(a,b) display the time evolution for the bipolaron’s mean charge density and bond-length, respectively, in an AGNR–4 with 300 Å of length with periodic boundary conditions, considering an electric field strength of 1.0 mV/Å applied in the direction of the armchair length. Figure 3 stands out the transport mechanism that defines the quasiparticle dynamics in organic semiconducting materials. It is evident in this figure that local charge density and lattice deformations associated with the presence of a bipolaron evolve together in time. Each strip in this figure represents a different time step. The first strip presents the initial state configuration for the charge localization (Fig. 3(a)) and the bond-lengths (Fig. 3(b)). As the time runs, we note that both charge density and bond-length present a collective motion toward the direction of the applied field. Initially, there is a delay in the bipolaron respond to the applied electric field. Such a waiting time for its motion derives from the way of turning on the electric field. Its strength is smoothly turned on, up to full value, to avoid the bipolaron destabilization, according to the procedure presented in ref.^27^. After a transient time, the bipolaron reaches a steady-state moving linearly through the lattice. Such a motion is evidenced by the changes in the vertical position of the charge density localization from the left to the right in the figure. The electric field plays the role of assisting the charge localization of a stable quasiparticle that presents a collective behavior during the charge transport. In the case depicted in Fig. 3, the bipolaron reaches its saturation velocity at about 100 fs. Indeed, the bipolaron dynamics in AGNRs occurs similarly to the polaron and bipolaron dynamics in conducting polymers, for instance^28^. This fact is another evidence that the formation of bipolarons can take place in GNRs.

**Figure 4:**
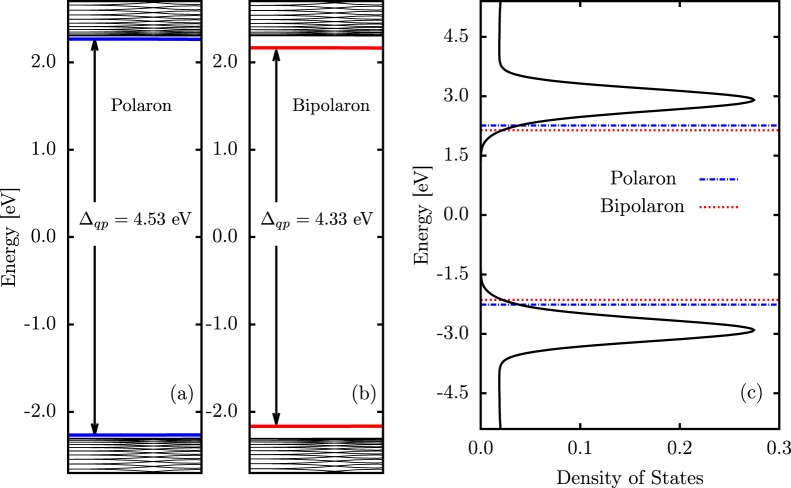
(**a**) Polaron energy levels, (**b**) bipolaron energy levels, and (**c**) the system’s density of states.

Polarons and bipolarons can also be distinguished in GNRs by analyzing the system’s electronic structure, as displayed in Fig. 4. In this perspective, Fig. 4(a,b) show the polaron and bipolaron energy levels, respectively, for a GNR–4 lattices. We can note that there are two levels — blue levels for the polaron and red levels for the bipolaron — within the bandgap. In these profiles for the energy levels, a bipolaron is denoted by a pair of states deeper inside the bandgap when compared to those of a polaron. The highlighted HOMO (Highest Occupied Molecular Orbital) and LUMO (Lowest Unoccupied Molecular Orbital) levels in Fig. 4 define a quasiparticle energy gap (Δ_*qp*_) that is 4.43 eV for the polaron and 4.33 eV for the bipolaron. In organic materials, smaller Δ_*qp*_ usually refers to more stable quasiparticles. Figure 4(c) presents the density of states (DOS) for the neutral lattice, that is both quantitatively and qualitatively similar to the DOS for systems containing one of these charge carriers. The dashed lines are referring to the Δ_*qp*_ for the polaron (blue lines) and bipolaron (red lines). In this particular framework for a charged GNR lattice, the presence of just one quasiparticle does not cause a substantial change in the bandgap value when compared to neutral systems.

**Figure 5:**
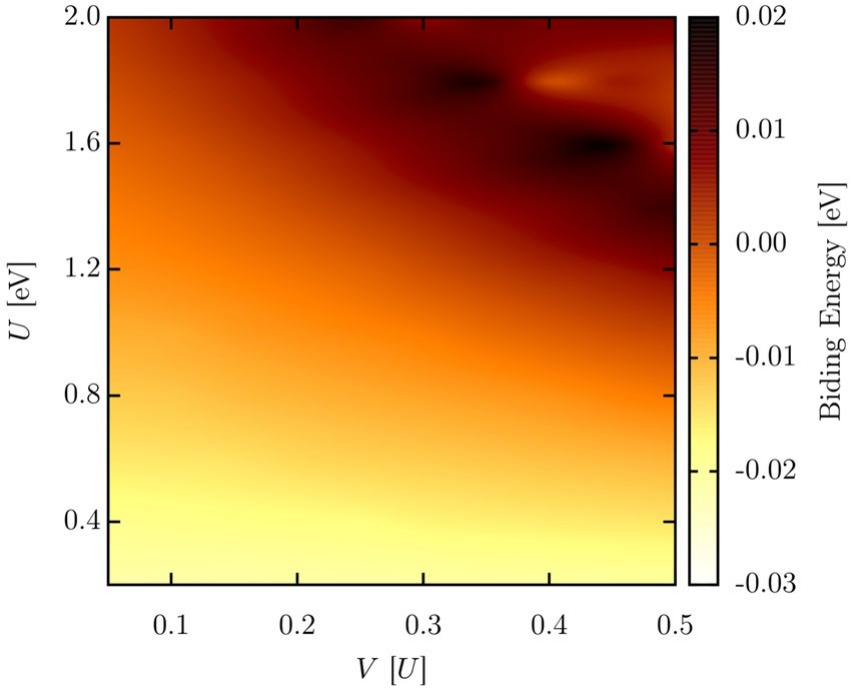
System’s binding energy (BE) as a function of the interplay between *U* and *V* (*V* has units of *U*).

To quantitatively characterize the bipolaron stability, we calculate the system’s binding energy (BE), i. e., the excess of energy required to couple the charge and lattice. Here, we define BE as *BE* = *ε*_*BP*_ − 2 × *ε*_*P*_, where *ε*_*BP*_ and *ε*_*P*_ are the bipolaron and polaron formation energies, respectively. The formation energy of a quasiparticle represents the difference between the energies for systems in the neutral ground state and a relaxed configuration due to the presence of an extra electron or hole. We analyze BE by considering the interplay between the intra (*U*) and inter-site (*V*) electron-electron interactions. According to the equation above, BE < 0 denotes a particular parameter space in which the energy required to obtain a stable quasiparticle is smaller in the formation of a bipolaron than two polarons. Therefore, for this case, bipolarons are the natural solution. Conversely, for BE > 0 the most convenient energetic solution for the system is in form of two isolated polarons. Based on this scenario for the BE interpretation, Fig. 5 illustrates the calculated BE as a function of the interplay between *U* and *V*. In this figure, *U* ranges from 0.2 to 2.0 eV, with step size of 0.2 eV, whereas *V* spans from 0.05 to 0.5 × *U* with increment of 0.05 × *U* (*V* has units of *U*). Figure 5 clearly shows that for *U* < 1.0 eV, for any strength of *V*, the BE is smaller than 0. It means that the self-consistent calculations for the energy minimization procedure always yields stable bipolarons as the solution. In these cases, the electronic repulsion is not strong enough to overcome the energy barrier imposed by the local lattice deformation to the extra holes that remain locally trapped and coupled to each other by forming a bipolaron. On the other hand, for *U* > 1.6 eV, considering all strengths of *V*, the BE always present positive values. For this parameter space, the electronic energy associated to the charge repulsion overcomes the lattice energy related to the local deformations in a such a way that the extra holes are actively repealed until a suitable distance in which their interaction can be neglected. In this physical picture, the separation between the additional charges yields two local lattice deformations coupled to the electronic state forming isolated (noninteracting) polarons. For *U* values between 1.0–1.6 eV, bipolarons can be created for small strengths of *V*, as can be inferred from Fig. 5.

**Figure 6:**
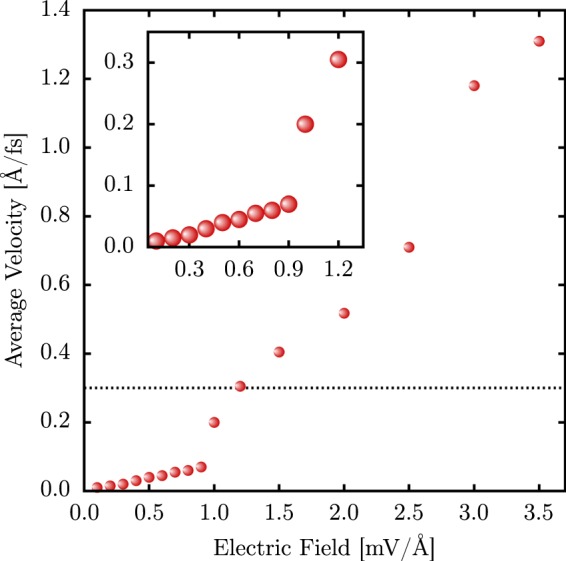
Interplay between the average bipolaron velocity and the strength of the applied electric field for an AGNR–4.

Finally, we turn to the role played by the electric field strength on the bipolaron transport mechanism. Figure 6 shows the behavior of the bipolaron average velocity as a function of the field strength. We systematically change the field strength within the intervals 0–1.0 and 1.0–3.5 mV/Å with an increment of 0.1 and 0.5 mV/Å, respectively. In this figure, the horizontal dotted line denotes the speed of sound in the material, approximately 0.3 Å/fs^14^. One can conclude that there are two distinct regimes for the bipolaron motion: the subsonic (acoustic) and the supersonic (optical) regimes. The phase transition between them occurs at 1.0 mV/Å. Strikingly, the bipolaron velocity, in this case, is equivalent to the speed of sound in AGNRs. At both transport regimes, the bipolaron velocity increases linearly by increasing the field strength. The abrupt changing in the slope of the two distinct lines refers to the bipolaron’s effective mass. Since the electric field plays the role of localizing the charge, there is a critical field strength, in this case, 1.0 mV/Å, for which the charge density for the bipolaron reaches its localization maximum. Such a kind of localization reduces the bipolaron effective mass by reducing the number of lattice distortions coupled to the charge that should be transported during its motion.

## Methods

Our two-dimensional tight-binding model is a modified version of the of the Su-Schrieffer-Heeger Hamiltonian^29,30^ that has the overall form *H* = *H*_*tb*_ + *H*_*ee*_, where

1 $${H}_{tb}=-\sum _{ < i,j > ,s}({t}_{i,j}{C}_{i,s}^{\dagger }{C}_{j,s}+h\mathrm{.}c\mathrm{.})+\frac{1}{2}(\sum _{ < i,j > }K{y}_{i,j}^{2}+\sum _{i}\frac{{P}_{i}^{2}}{M})$$

and

2 $${H}_{ee}=U\sum _{i}({C}_{i,\uparrow }^{\dagger }{C}_{i,\uparrow }-\frac{1}{2})({C}_{i,\downarrow }^{\dagger }{C}_{i,\downarrow }-\frac{1}{2})+V\sum _{\langle i,j\rangle }({n}_{i}-1)({n}_{j}-1)\mathrm{.}$$

**Figure 7:**
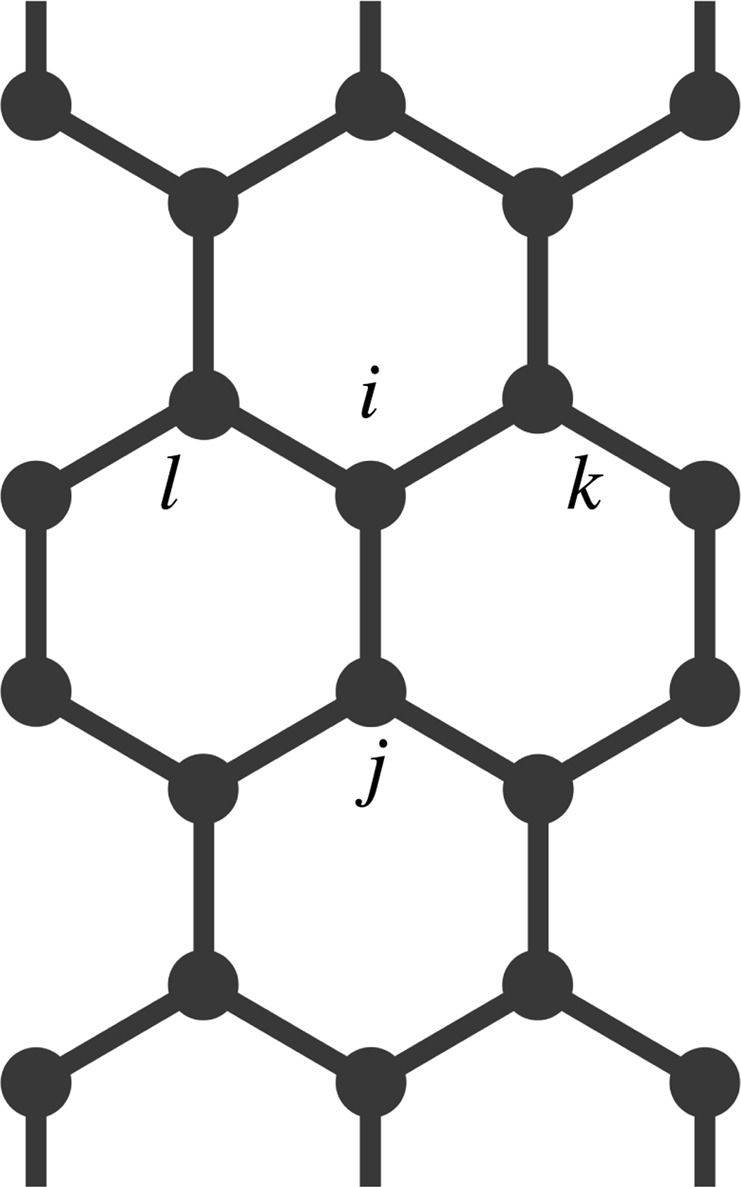
Schematic representation of an armchair GNR. The site labeled as *i* has tree neighbors: *j*, *k*, and *l*.

In Equation 1, the indexes *i* and *j* denote two arbitrary neighboring sites in the lattice (Fig. 7). The bond length for two such sites is *y*_*i*,*j*_, *t*_*i*,*j*_ stands for the hopping integral of a *π*-electron between nearest-neighboring sites, which assumes the following form

3 $${t}_{i,j}={e}^{-i\gamma {\bf{A}}({\bf{t}})}({t}_{0}-\alpha {y}_{i,j}\mathrm{).}$$

In the equation above, the first part describe the electronic contribution. The exponential term denotes a Peielrs substitution on the phase factor to include a time-dependent vector potential^27^. Through the potential vector, we can consider a time-dependent electric field by using **E**(t) = (−1/c)$\dot{{\bf{A}}}$(t). $\gamma \equiv ea/\hslash c$, where *a* is the lattice parameter, *e* is the absolute value of the electronic charge, and *c* is the speed of light. *t*_0_ is the hopping integral for a neutral lattice in which the carbon atoms are equally spaced and *α* is the electron–lattice coupling constant that couples the two distinct degrees of freedom, i.e., the electronic and lattice contributions. ${C}_{i,s}^{\dagger }$ (${C}_{j,s}$) creates (annihilates) a *π*-electron with spin *s* in the *i*-th (*j*-th) site. The second part in Equation 1 and the expectation value of the fisrt part govern the lattice description. Such degree of freedom is addressed in the context of a harmonic approximation to account for, effectively, the potential related to the *σ* bonds. In this way, *K* is the force constant. *P*_*i*_ is the conjugated momentum of a carbon atom, and M is its mass. We include electron-electron interactions within an extended Hubbard formalism, as written in Equation 2. In that expression ${n}_{i}={C}_{i,\uparrow }^{\dagger }{C}_{i,\uparrow }+{C}_{i,\downarrow }^{\dagger }{C}_{i,\downarrow }$. *U* corresponds to the on-site electron–electron coulombic interaction, and *V* is the neighboring sites electron–electron interactions.

It is worthwhile to stress here that the initial system configuration contains a bipolaron in its stationary state arrangement. To achieve such a initial picture, we use the self-consistent procedure described in ref.^27^ that was employed to obtain stable polarons in armchair GNRs. However, here, our numerical protocol consists in removing from the lattice two electrons with antiparallel spins to create a positive bipolaron. The time evolution of the system — that considers the coupled electronic and lattice degrees of freedom — is governed by an Ehrenfest Molecular Dynamics approach, according to ref.^27^. Importantly, the employed parameters for the model Hamiltonian are: *t*_0_ = 2.7 eV, *M* is the carbon core’s mass, *K* = 21 eV/Å^2^, *α* = 4.1 eV/Å, and *a* = 1.44 Å. This set of parameters was successfully used in other theoretical works based on SSH-like models^14,15,17,20,27,31^.

## Conclusions

In summary, the formation and dynamics of bipolarons in armchair graphene nanoribbons were numerically studied in the framework of a two-dimensional tight-binding approach that includes electron-lattice interactions. Our findings have shown that bipolarons are possible quasiparticle solutions when it comes to AGNRs. The charge carrier dynamics under an external electric field revealed that these quasiparticles are stable structures and can move within two distinct regimes concerning their saturation velocity. In the first regime, a bipolaron moves through the material with an average velocity that is lower than the speed of sound, i. e., an acoustic (subsonic) transport mechanism. In the second one, the bipolaron average velocities are higher than this critical value, which shows an optical (supersonic) regime for the carrier dynamics. Interestingly, for 1.2 mV/Å, the bipolarons moves roughly with the speed of sound in the material. The phase transition between these two transport regimes occurs at 1.0 mV/Å and two linear trends, with different slopes, represent the interplay between the field strength and the bipolaron saturation velocity.
